# Ketogenic Diet and Endocrine and Metabolic Diseases: A Bibliometric Study and Visualization Analysis

**DOI:** 10.2174/0118715303317289240820114329

**Published:** 2024-10-28

**Authors:** Xinwei Zhang, Yanfei Jiang, Tiantian Cai, Zhaowei Huang, Yuqing Wu, Jinan Zhang

**Affiliations:** 1 Graduate School, Shanghai University of Traditional Chinese Medicine, Shanghai, 201203, China;; 2 Department of Endocrinology and Rheumatology, Shanghai University of Medicine and Health Sciences Affiliated Zhoupu Hospital, Shanghai, 201318, People’s Republic of China;; 3 Shanghai University of Traditional Chinese Medicine, Shanghai, 201203, People’s Republic of China

**Keywords:** Ketogenic diet, endocrine and metabolic diseases, VOSviewer, bibliometric, CiteSpace, 3-β-hydroxybutyrate

## Abstract

**Background:**

The ketogenic diet, known for its high-fat, low-carbohydrate composition, has been extensively studied in endocrine and metabolic diseases. This study carried out bibliometric analysis to examine the research trends in this field over the past 20 years, aiming to provide insights for future studies.

**Methods:**

We searched the Web of Science Core Collection for all relevant papers. VOSviewer was used for network visualization, the bibliometrix package of R software (version 4.3.0) was utilized for data analysis, and CiteSpace was employed for mapping and trend analysis.

**Results:**

This study encompassed 508 relevant articles spanning from 2003 to 2023, authored by 2827 researchers from 887 institutions across 57 countries/regions. The total number of publications increased from 3 in 2003 to 508 in 2023, showing a steady growth trend. The United States emerged as the predominant contributor in this field, followed by Italy and China. Notably, SAJOUX I consistently exhibited high activity in this field, according to the analysis, with an h-index of 13. The journal *Nutrients* has consistently made substantial contributions to this field, accounting for 19% of all publications. The keywords “obesity,” “ketogenic diet,” and “weight loss” appeared most frequently, with “obesity” occurring 163 times.

**Conclusion:**

This study used a bibliometric method to analyze the impact of the ketogenic diet on the endocrine metabolic system. The research identifies recent frontiers and trending directions, providing valuable references for scholars in this field.

## 1 INTRODUCTION

The Ketone Bodies (KBs) are intermediate metabolites in the oxidative metabolism of fats. They are composed of 3-β-hydroxybutyrate (βOHB), acetoacetate, and acetone. These molecules are present in large quantities in the bloodstream, while some can be excreted in urine, breath, and sweat [1]. KBs serve as a source of energy for the tissues during fasting or prolonged exercise [2]. The ketogenic diet (KD) is a therapeutic diet that is isocaloric, high in fat, low in carbohydrates and contains moderate amounts of protein and other nutrients [3, 4]. KD was initially developed to mimic the biochemical effects of intermittent fasting on the body [5]. The KD model utilizes fat as the body's primary fuel source, shifting energy metabolism from glucose to KBs produced by the liver. The classic KD typically consists of a ratio of 3:1 or 4:1 of fat to carbohydrates and protein (in grams) [6]. However, due to the large number of adverse effects of the classic KD, there are currently four major KDs that have been improved: the classic KD, the modified atkins diet (MAD), the medium-chain triglyceride diet (MCT), and the low glycemic index treatment (LGIT) [7]. Initially, the KD was mainly utilized to treat medically refractory epilepsy. However, it now has applications in treating various ailments, such as cancer, migraines, diabetes, and obesity [8-10].

Chronic endocrine and metabolic diseases represent a significant global health burden, affecting millions and significantly impacting international public health. The development of these diseases is primarily influenced by the interaction between genetic and environmental factors, including lifestyle and dietary patterns. Endocrine and metabolic disorders often affect the functioning of multiple organ systems and lead to abnormalities in carbohydrate, protein, fat, water, electrolyte, and purine metabolism, all of which are closely related to our dietary intake. Despite advancements in modern medicine and a range of treatments, from pharmacological therapies to lifestyle adjustments, managing these complex conditions remains a challenge, underscoring the need for continued research into effective interventions. Recently, the focus on dietary patterns as a critical component of prevention and management has deepened, reflecting their pivotal role in influencing these disorders. For example, a study has found a potential impact of dietary patterns on insulin resistance and overweight [11]. Lifestyle and dietary improvements are among the preventive and therapeutic measures for diabetes. KD, as a non-pharmacological treatment approach and one of the dietary patterns, has gained attention from medical experts. Obesity is a primary risk factor for various metabolic disorders [12]. Animal and clinical experiments in obesity models have shown that ketogenic dietary interventions improve some metabolic indicators [13, 14].

Therefore, our attention is now turning to the interaction between KD and endocrine-metabolic system disorders. To understand the forefront and hot topics in the research literature, the most common approach is to conduct a bibliometric analysis. Despite the prevalence of review articles on the KD, which provide insights into its efficacy for specific diseases, there remains a lack of systematic, quantitative bibliometric analyses to comprehensively map the research landscape in this field. This is particularly true for its applications in endocrine and metabolic disorders. A thorough bibliometric analysis would not only reveal global research trends and key contributors but also identify potential areas that require further exploration, thus supplementing and expanding upon areas not covered by existing individual studies and reviews. Bibliometrics, as an interdisciplinary field, employs mathematical and statistical methods to quantitatively describe scholarly information and conduct statistical analyses. It is an effective method for visualizing and analyzing research status and trends. Bibliometrics was first proposed by Alan Pritchard in 1969 and is now applied in various disciplines [15]. Bibliometric analysis can analyze existing publications and investigate specific information, such as countries or regions, institutions, journals, and authors, in order to assess the research capacity of authors and national institutions in the field. This analysis can also identify contributions and rankings of relevant topics under multiple influencing factors and provide references and insights for subsequent research in this area. To assess the breadth and depth of research on the KD within the field of endocrine and metabolic diseases, this study aims to quantitatively delineate the research trends and historical progression in this domain. By analyzing the volume of published literature and the diversity of research topics, we seek to uncover the overarching trajectories and thematic evolutions. Through these objectives, this research tries to fill the existing gaps in the systematic and quantitative analysis of KD applications in endocrine and metabolic disorders, providing a comprehensive perspective and in-depth insights into the field.

## 2 METHODS

### 2.1 Data Source and Search Strategy

**Fig. (1):**
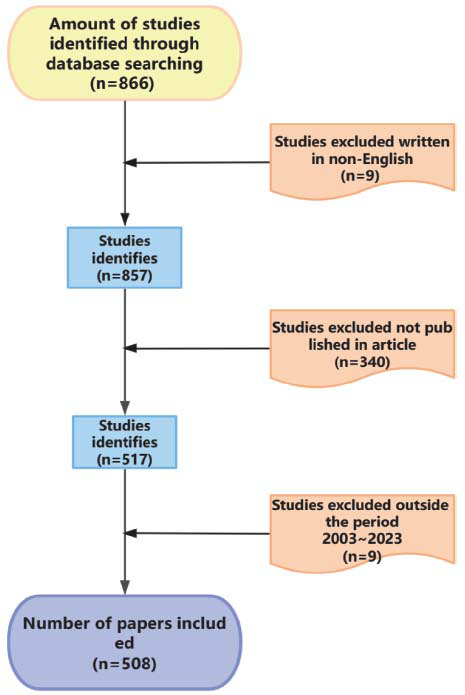
The flowchart on the methodology and the process used in this study.

All data in this article were retrieved from the Web of Science Core Collection (WoSCC) database on September 22^nd^, 2023, and all the search was completed on the same day to avoid bias in the number of documents due to database updates. Synonyms of search terms and related disease terms were used to expand the search and ensure a comprehensive search, with the following search pattern: (((TS=(Diet, Ketogenic)) OR TS=(Ketogenic Diet)) OR TS=(Diets, Ketogenic)) OR TS=(Ketogenic Diets) AND (((((((((((TS=(Endocrine System Diseases)) OR TS=(Endocrine Disease)) OR TS=(Diabetes Mellitus)) OR TS=(obesity)) OR TS=(non-alcoholic fatty liver disease)) OR TS=(gout)) OR TS=(Polycystic Ovary Syndrome)) OR TS=(Graves Disease)) OR TS=(hypothyroidism)) OR TS=(Cushing Syndrome)) OR TS=(Hypercortisolism)) OR TS=(Hyperlipidemias). A total of 866 papers were retrieved. Nine non-English language papers were excluded, along with 340 non-article type papers, and nine papers outside the 2003 to 2023 interval were also excluded. Finally, 508 publications were included in the bibliometric analysis. All relevant publications, including titles, authors, keywords, institutions, nations, references, and citations, were saved and exported as plain text files (Fig. **1**).

### 2.2 Data Analysis And Visualization

The selected data was visualized and analyzed using software and websites, such as VOSviewer, R package Bibliometric, CiteSpace, and ProcessOn. VOSviewer version 1.6.19 was used to display keyword co-occurrence networks and conduct cluster analysis. CiteSpace version 6.2.6 was employed to show a dual-map overlay of journals and burst term maps. Time slices were set annually to track the development dynamics of the field. The R language version 4.3.0 with bibliometrix package version 4.1.2 was used as a supplementary tool for the main visualization analysis, depicting three-field plots for authors, keywords, and journals, as well as keyword clouds, countries, and journals. The online tool ProcessOn (https://www.processon.com/) was used to create flowcharts illustrating the search process. Microsoft Office Excel 2021 was employed to visualize the number of publications and annual trends.

## 3 RESULTS

### 3.1 The Trend of Global Publications And Citations

**Fig. (2):**
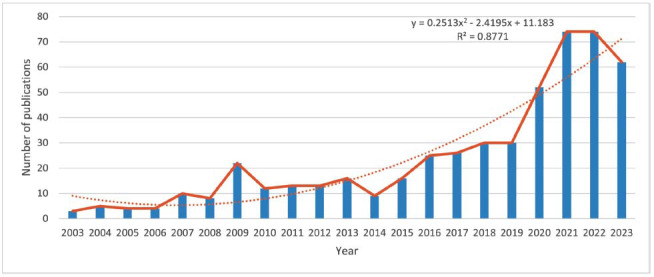
Annual growth trend of publications.

A total of 508 articles on ketogenic diet and endocrine system disorders were included in this study, and the number of papers provided a general indication of the research hotspots and directions of the related topics, as shown in (Fig. **2**), which shows an overall increasing trend from 2003 to 2023. A regression model was constructed using a polynomial with the equation y = 0.2513x^2^ - 2.4195x + 11.183. Exponential fitting was a method of fitting the ketogenic diet to the endocrine system based on an exponential function fitting method, which was used to characterize the growing trend in the data. There was a positive correlation between the number of papers published per year and the year of publication (R^2^= 0.8771). It can be roughly categorized into two phases. The number of publications from 2003 to 2009 was in a period of continuous growth, but the number of publications during this period was still relatively small. The overall growth from 2010 to 2023 was faster, especially in 2021 and 2022, and the number of publications in 2023 was not too high, probably because of the incomplete entry of the data, which happened before September. Papers published in 2004 and 2010 showed a strong impact, as mentioned in Table **1**, with the top five total citations being Yancy (2004), Dushay (2010), Hopkins (2018), Kennedy (2007), and badman (2009). This result suggests that the role of the KD in endocrine system disorders has gained increasing attention in recent years and is becoming a hot research topic.

### 3.2 Analysis of Countries/Regions And Institutions

**Fig. (3):**
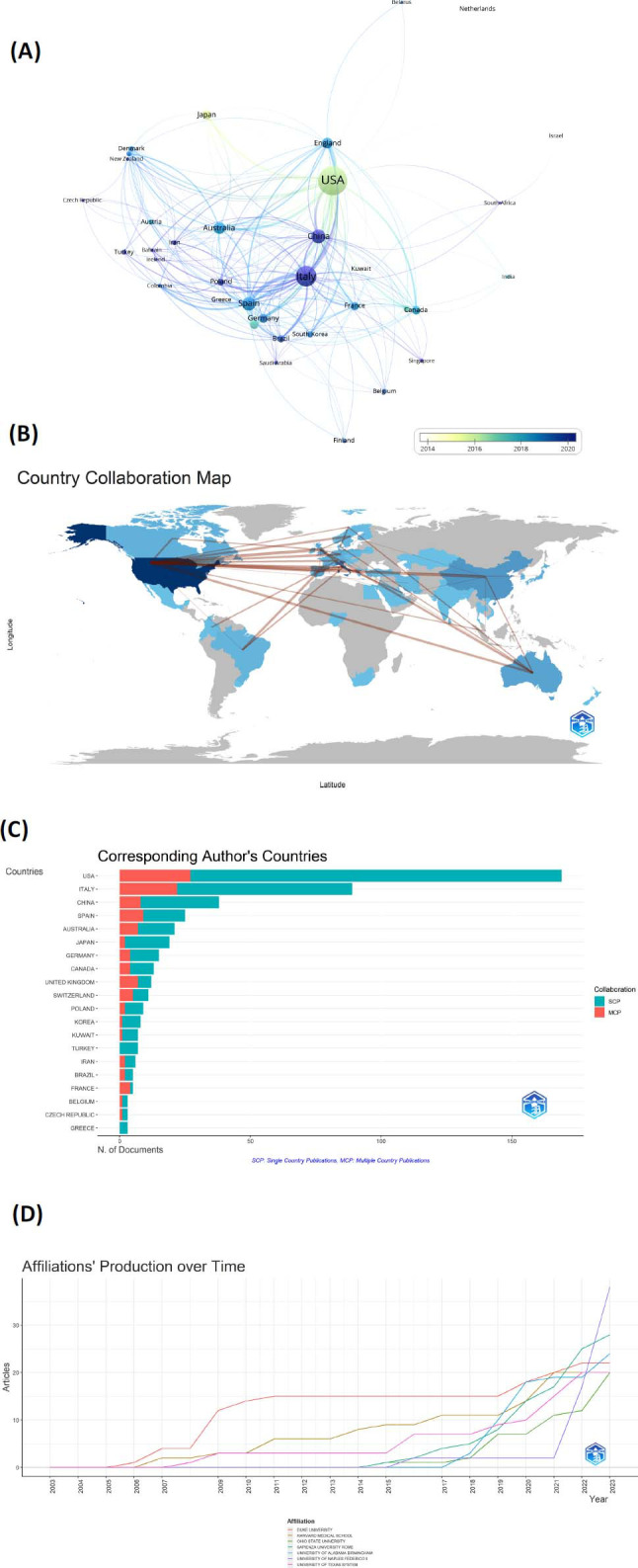
**(A)** Visualization of national collaboration. **(B)** Collaborative geographic maps of different countries/ regions. **(C)** Ranked according to the country of the top 20 corresponding authors of the MCP/SCP. **(D)** Institutions’ production from 2003 to 2023.

According to the analysis of WoSCC data, 57 countries/regions and 887 institutions have been involved in research on related topics in the last 20 years. Table **2** presents the top 10 contributions of countries and institutions, with the United States in the first place with 169 publications, followed by Italy and China in the second and third places with 89 and 38 publications, respectively. The two core clusters in the country collaboration were the United States and Italy (Fig. **3A**). The geographic visualization of national outputs and collaborations analyzed in the bibliometrix R package showed that the research carried out was mainly concentrated in North America, Europe, Asia, and Australia (Fig. **3B**). It is worth noting that different countries have directly engaged in active collaboration, with the connecting lines between countries indicating previous collaborative relationships between countries, with North America engaging in more collaborations with Europe. Among all the countries, the USA had the highest percentage of SCPs (169 articles in total; SCP:142; MCP:27, MCP-Ratio: 0.16). Italy has an SCP of 67, and China is the third with an SCP of 30, with 38 papers (Fig. **3C**).

In terms of ranking of publications, the top 10 institutions are mentioned in Table **2**, with the University of Naples Federico II (38 publications) being the most productive, followed by Sapienza University Rome (28 publications), University Of Alabama Birmingham (24 publications), and Duke University (22 publications). (Fig. **3D**) shows that the University Of Naples Federico I was marked by rapid growth in 2021, and from that year onwards, the number of articles on the school has grown rapidly, directly outstripping the rest of the research institutions.

### 3.3 Author Analysis

**Fig. (4):**
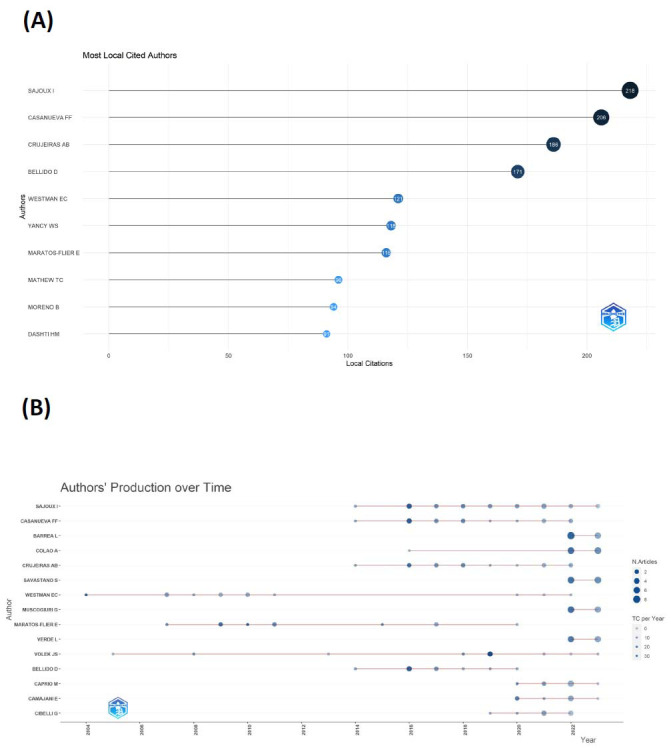
**(A)** Top 10 of most locally cited authors. **(B)** The number of articles produced by the top 15 authors varied over time.

Analysis of the acquired data revealed that over the past two decades, a total of 2,827 authors have made contributions to the study of the correlation between KD and endocrine system disorders. H-index can be used to assess the amount of scholarly output and the level of scholarly output of researchers [16]. The G-index is mainly proposed to compensate for the shortcomings of the h-index, which does not respond well to highly cited papers [17]. The M index is an author’s H index/the years since their first publication [18]. The figure illustrates collaborative ties among authors, and Table **3** outlines the top ten authors under various impact indices (h-Index, g-Index, m-Index). Notably, SAJOUX I (h-index: 13, g-index: 19, m-index: 1.3) stands out as the most influential figure in this field, securing the top spot among locally cited authors (Fig. **4A**). Additionally, the graph highlights that WESTMAN EC embarked on research in this domain as early as 2004, with a considerable duration and a total citation count of 1,271 (Fig. **4B**). The majority of papers were published in 2020. MARATOS-FLIER E emerged as the most cited author, boasting an h-index of 10, g-index of 10, and m-index of 0.58, underscoring their substantial impact in the literature of this research field.

### 3.4 Journals Analysis

**Fig. (5):**
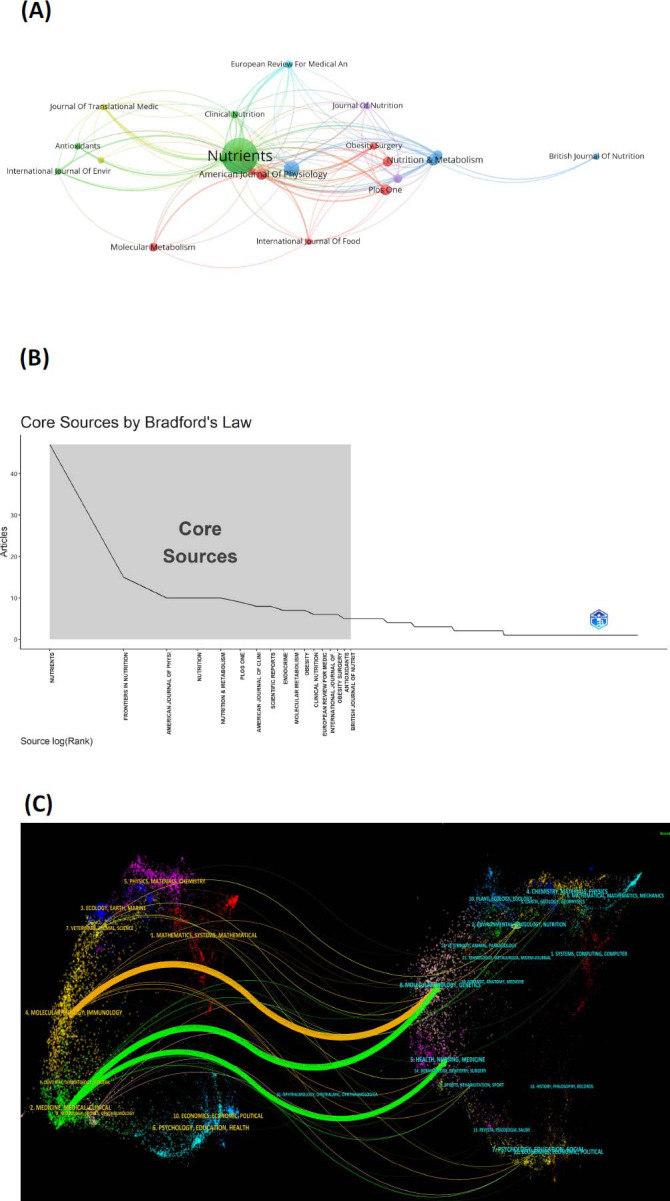
**(A)** The co-cited journals network visualization. **(B)** Analysis of journals based on Bradford's Law. **(C)** The dual-map overlay of journals on the research of KD in endocrine and metabolic diseases.

Research articles on the subject have been published in 253 journals, with the top five in terms of publication volume being Nutrients (IF: 5.9), Frontiers in Nutrition (IF: 5), American Journal of Physiology-Endocrinology and Metabolism (IF: 5.1), and Nutrition (IF: 4.4). Journal impact factor (IF) serves as a quantifiable metric of a journal's influence and stands as a crucial indicator for assessing academic productivity within the scholarly community. This data collectively indicates a particular enthusiasm within these journals for research related to KD and endocrine metabolic disorders (Fig. **5A**).

Bradford's law stands as a fundamental principle in bibliometrics, primarily elucidating the relationship between journals and the number of published papers (Fig. **5B**). It asserts that scientific journals within a specific field can be categorized into core and subsequent zones based on the number of publications, adhering to a ratio of 1:n:n^2^ [19]. Journals analyzed through the lens of this law predominantly cluster within JCR Q1 and Q2, indicating a higher quality of journals in the data analysis conducted for this study Table **4**.

Through CiteSpace, we conducted a dual-map overlay analysis of journals, revealing the relationship between citing and cited references [20]. On the left side, citing journals are depicted, while the right side illustrates cited journals. Three primary citation pathways are discernible, delineated in orange and green (Fig. **5C**). The orange pathway, the primary citation route, signifies research published in molecular/biology/immunology journals, predominantly referenced by literature in molecular/biology/genetics and health/nursing/medicine publications. Both molecular/biology/genetics and health/nursing/medicine journals stand as commonly cited works within medicine/medical/clinical journals.

### 3.5 Analysis of High-frequency Keywords

**Fig. (6):**
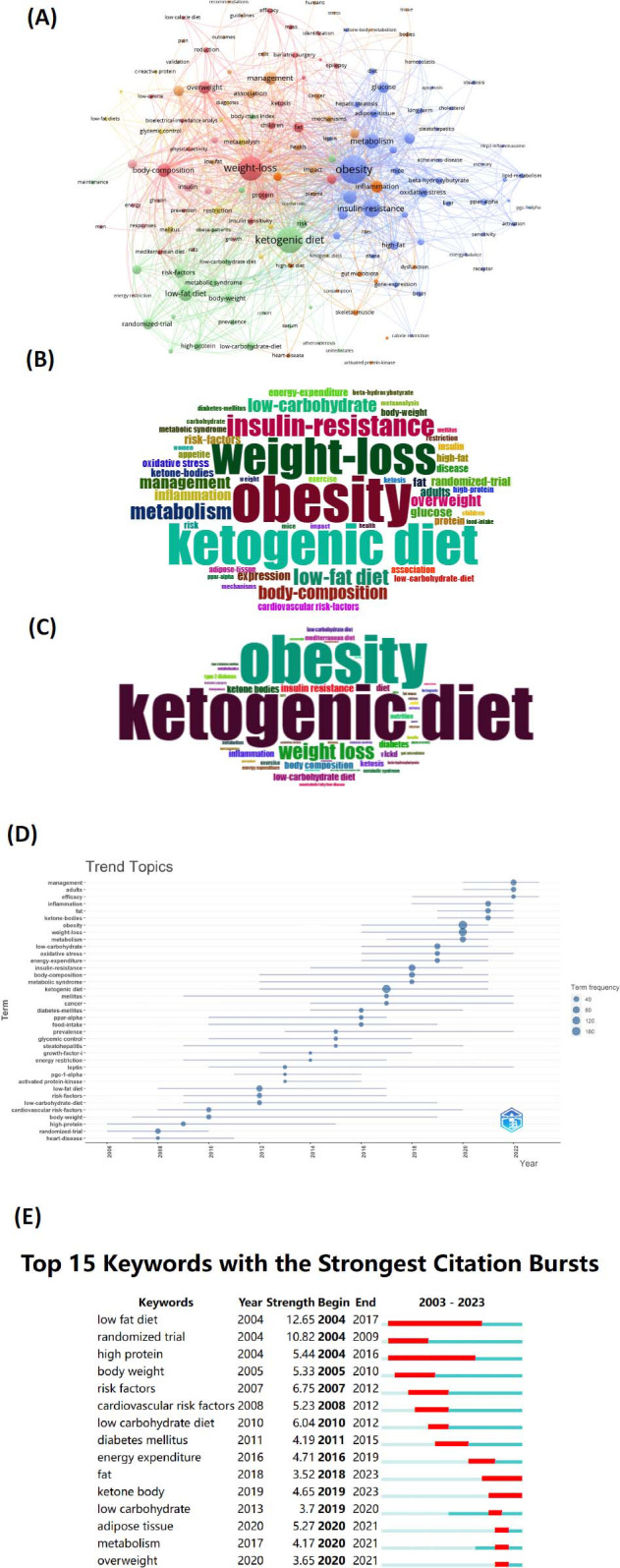
**(A)** The co-citation of keywords Plus generated by VOSviewer. **(B)** Word cloud of top 50 keywords plus. **(C)** Word cloud of top 50 authors’ keywords. **(D)** Author's keywords trend topics. **(E)** The top 15 word bursts of this topic according to CiteSpace.

To visually depict the high-frequency keywords in this field, both VOSviewer and CiteSpace were employed. A total of 150 entries emerged from all the keywords, organized into seven clusters (each keyword appearing at least five times). Different colors denote distinct research orientations and objectives. The most substantial cluster, Cluster 1 (purple), encompasses 43 elements; following prominently are Cluster 2 (green) and Cluster 3 (pink) (Fig. **6A**). Utilizing the bibliometrix R package, we generated a keyword cloud, revealing that “obesity,” “weight loss,” and “ketogenic diet” were the most frequently occurring terms, aligning with the results from the VOSviewer diagram (Figs. **6B** and **6C**). Over time, an emerging trend in research focus has been observed, with inflammation and obesity gradually becoming prominent in the related field (Fig. **6D**). Employing CiteSpace for burst analysis allows for a more detailed exploration of research hotspots and frontier dynamics over specific periods, aiding in predicting future research trends [19]. The burst analysis diagram spanning 2004 to 2017 highlights a “low-fat diet” and “high protein”. Notably, from 2019 to 2021, the burst terms predominantly include “low carbohydrate,” “fat,” “metabolism,” and “overweight,” indicating an increasing exploration of metabolic disorders (Fig. **6E**). It shows key high-frequency terms, such as “obesity,” “ketogenic diet,” “weight loss,” “insulin resistance,” “low-fat diet,” and “metabolism,” suggesting that these research directions remained popular and may represent future research developments Table **5**.

## 4 DISCUSSION

With the changes in people’s lifestyles and the intensification of global aging, the incidence and prevalence of many endocrine and metabolic system diseases are gradually increasing. Although pharmacological treatments are the dominant approach, they may have adverse effects on the organism. Therefore, non-pharmacological interventions, such as dietary modifications, have emerged as an effective means of assisting in the treatment of these disorders. The KD, a dietary pattern that combines a very low intake of carbohydrates with a high intake of fats, offers a new perspective on the treatment of a wide range of diseases by inducing the metabolism of body fat and the production of KBs as an alternative to traditional sources of energy. These KBs are distributed to metabolically active tissues and organs, absorbed by monocarboxylate transporters (MCTs) in extrahepatic tissues, converted to acetyl-CoA, and ultimately oxidized in the tricarboxylic acid cycle [2]. This dietary approach affects several biochemical processes and improves the clinical management of various diseases, such as obesity [21], type 2 diabetes [13], polycystic ovary syndrome [22], non-alcoholic fatty liver disease [23], gout [24], Cushing’s syndrome [25], and others.

This study analyzed 508 research articles from the Web of Science using various visualization software, such as VOSviewer, R software (version 4.3.0) bibliometrix package, and CiteSpace. The number of articles on the KD and its relation to endocrine and metabolic diseases steadily increased from 2010 to 2023. In the past two years, the number of articles published has remained at about 74, indicating a growing interest in this field of research on an international level. A total of 57 countries/regions, 887 institutions, and 2827 authors participated in publishing literature in this field. Among them, in terms of countries, the United States has the world's highest contribution to the study of topics in this field, with 89 articles in the first place, followed by Italy and China. From the country geographic collaboration map, it can be seen that North America, Europe, Asia, and Australia cooperate more closely. At the same time, most of the top 10 institutions are also mainly from the United States, Italy, and France, with the most representative being the University of Naples Federico II, Sapienza University Rome, the University of Alabama Birmingham, and other colleges and universities, indicating that the universities in the United States and Italy have high academic influence in this field. The most prolific journals in this field are Nutrients, Frontiers in Nutrition, and the American Journal of Physiology-Endocrinology and Metabolism. Highly cited literature reflects the current research trends. In 2004, William S. Yancy Jr. *et al.* published a paper titled “A Low-Carbohydrate, Ketogenic Diet *versus* a Low-Fat Diet to Treat Obesity and Hyperlipidemia: A Randomized, Controlled Trial” [26]. It compared the effects of a KD and a low-fat diet on obesity and hyperlipidemia, confirming the advantages of the KD in regulating blood lipids.

In many incorporated works of literature, with the continuous improvement of the form and content of the KD, it can fetch some results in several common diseases, such as diabetes mellitus, obesity, non-alcoholic fatty liver disease, and polycystic ovary syndrome. Diabetes mellitus is a common endocrine and metabolic disease, often associated with several complications, and has been a common and serious health problem worldwide. The high prevalence of diabetes is usually closely related to a number of risk factors, such as unhealthy diet and lifestyle [27]. Alaa Al-Khalifa's team studied the role of low-carbohydrate KD in diabetes. They found that this dietary pattern can reduce blood glucose levels after STZ injections in the short term and control the lack of weight loss. Glucose levels in the urine appeared high in the early stages but almost normal in the later stages [28]. Application of a ketogenic VLCD reduces glycated hemoglobin levels in adolescents with type 2 diabetes mellitus [29]. Yuan *et al.* conducted a systematic review and meta-analysis and reported that KD reduces BMI and body weight, increases insulin sensitivity and glycemic control, and improves glucose-lipid metabolism in patients with type 2 diabetes mellitus [30]. GLT2i medications, when used to treat type 2 diabetes, induce a state of ketosis that helps reduce cardiovascular mortality and the risk of hospitalization for heart failure, demonstrating the potential benefits of ketones, as seen in KD [31, 32]. Additionally, studies suggest that KDs may modulate the physiological response to hypoglycemia, potentially by providing alternative energy sources, such as ketones, to delay the activation of the sympathetic nervous system, thereby protecting the body during times of insufficient glucose supply [33]. Similarly, obesity, as the first of the keywords, is a symptom that accompanies the presence of many metabolic diseases. Obesity rates are on the rise in most countries of the world, and with the accelerated industrialization and urbanization of countries, changes in diets and lifestyles lead to weight gain, increasing the risk of other cardiovascular diseases and cancers and ultimately affecting health [34, 35].The KD for weight control management of obesity is mainly achieved through two modified ketogenic dietary patterns: the very low-calorie ketogenic diet (VLCKD) and the low-calorie ketogenic diet (LCKD) [36-38]. Andrea Deledda *et al.* applied VLCKD in combination with the Mediterranean diet to study patients with type 2 diabetes mellitus and obesity. They found that the VLCKD treatment may provide greater benefits than the traditional Mediterranean diet in the short term, particularly in the areas of weight, body mass index (BMI), body fat percentage (FM%), and waist circumference (WC) [39]. Obesity is often accompanied by inflammatory processes as well, and Luigi Barrea *et al.* found significant reductions in body weight as well as inflammatory levels, significant decreases in hs-CRP, and elevated PhA in obese and primiparous hypertensive female patients after VLCKD treatment [40, 41]. The liver is the primary site for the production of bile acids (BA), KB, and lipids [42]. Non-alcoholic fatty liver disease (NAFLD) is typically identified by a range of fibrosis, from hepatic steatosis to non-alcoholic steatohepatitis (NASH), which may ultimately result in cirrhosis and hepatocellular carcinoma [43]. Alexa N King *et al.* found that administering the KD through an HFS-induced NAFLD rat model maintained insulin sensitivity and inhibited lipid accumulation in the liver [44]. The KD enhances fatty acid oxidation, resulting in reduced liver lipogenesis [45]. A study indicates that during a KD, changes in LDL-cholesterol can be predicted by body composition (such as body mass index, fat mass index, and lean mass index) as well as thyroid hormones, particularly free T3 and total T4 [46]. This suggests that the impact of KD on health risk factors is closely related to an individual's endocrine and metabolic state. By elevating ketone levels in the body, the KD can mimic the metabolic state of prolonged fasting. This dietary strategy is not limited to managing diabetes or obesity but has also been studied for the treatment of intractable epilepsy and other neurological disorders. The article emphasizes that ketones play a crucial role in suppressing inflammation and regulating immune responses, potentially by affecting post-translational modifications of proteins. PCOS, or polycystic ovarian syndrome, is a common endocrine condition that affects women who are fertile. Changes in metabolism, reproduction, and psychology are its defining characteristics [47]. Patients with PCOS are often hyperinsulinemic and hyperandrogenic. Lifestyle changes, such as interventions in diet and exercise, have a positive impact on PCOS [48]. Srdjan Pandurevic *et al.* conducted a randomized controlled trial administering a VLCKD to women with PCOS, with a Mediterranean low-calorie diet (LCD) as a control. The study found that the VLCKD group showed significant improvements in anthropometric and body composition parameters, metabolic and hormonal variables, and gynecological clinical outcomes, including menstrual abnormalities and pregnancy rates, as well as a reduction in visceral fat [49]. Additionally, the KD may improve the endocrine and metabolic conditions of PCOS patients by reducing insulin resistance and lowering blood sugar levels [50]. By controlling weight and fat content, it is possible to further improve patients' hormone levels and reproductive health. Although this dietary approach has shown some positive clinical effects, the evidence of its application in adolescents remains limited, necessitating further research to validate its long-term effects and safety. In a meta-analysis concerning the effects of the KD and the Dietary Approaches to Stop Hypertension (DASH) diet on serum uric acid levels, the DASH diet showed positive effects in reducing serum uric acid. In contrast, the KD had no significant impact on serum uric acid levels. Although there might be a temporary increase in uric acid levels at the beginning of ketosis, the long-term effects are not apparent [51]. Thus, the DASH diet might be a more favorable choice for managing hyperuricemia and preventing gout. Additionally, β-hydroxybutyrate (BHB), a ketone produced during the KD, has been shown to alleviate mitochondrial dysfunction caused by myocardial ischemia, thus improving cardiac contractility and structural remodeling [52]. Interestingly, in an editorial discussing the effects of endocrine modulators on pediatric neurological diseases, KD is highlighted for its potential positive effects on brain development and injury repair, particularly in the treatment of intractable epilepsy in children [53].

Burst keywords represent current research trends and potential developments in a given field. The study shows that obesity, KD, and weight loss are popular research topics in this field. Several researchers have confirmed that KD has a positive impact on managing endocrine metabolic diseases, such as obesity. In the future, it may be important to analyze the effects of a KD on endocrine metabolic diseases, such as obesity. Moreover, KD has significant clinical value as an adjunctive dietary treatment. Therefore, it is recommended that an in-depth study of its related mechanisms of action be conducted. Although several studies have shown that KD is a relatively safe dietary approach, most of these studies have reported short-term improvements, and there may be some adverse reactions and side effects in the long term, which requires further evaluation of the safety. Common side effects of KD include dizziness, fatigue, insomnia, palpitations, constipation, and muscle pain, both short-term and long-term [54, 55]. Long-term KD intake has been associated with the risk of kidney stones, gallstones, elevated liver enzymes, and, in rare cases, death [56]. Severe adverse reactions are associated with increased levels of ketones, which can cause an increase in redox imbalance and increase the incidence and mortality rates of diabetic patients with ketoacidosis [57]. Although the KD offers new possibilities for the treatment of a wide range of endocrine and metabolic disorders, its long-term effects and safety still require further research. Future studies should focus on how to optimize the formulation of the KD to reduce adverse effects and validate its effectiveness through more extensive clinical trials. In addition, understanding the specific mechanism of action of the KD in different populations will help to better individualize treatment regimens to maximize the value of its clinical application.

However, our study also has some limitations. Our study was based on bibliometric data, which inherently focuses more on the quantity of research findings and less on the quality or clinical validity of the reported findings. This limitation suggests that although KDs are frequently mentioned in academic articles, the actual efficacy and safety of these dietary interventions may not be as widely validated as our analysis suggests. There may be some bias in our analysis because studies with positive results are often more likely to be published than those with negative results, which may influence researchers' perceptions of the effectiveness of KD in treating endocrine and metabolic diseases. As our study focused primarily on articles and excluded article types, such as reviews, selecting articles based on keywords and indexing terms may have excluded relevant studies that were not appropriately labeled, which may have missed critical data that could have affected the overall analysis. Our study was confined to the past 20 years and may not have included some of the most recent or previously published relevant articles.

## CONCLUSION

In conclusion, our bibliometric analysis underscores the growing interest and research progression in the role of KD within endocrine and metabolic diseases over the past two decades. This paper provides a historical summary and current trends in research, highlighting the short-term therapeutic benefits of this diet on several disorders within this category. Despite these findings, it is crucial to acknowledge the limitations and potential biases inherent in our study. The mechanisms of action and side effects of the KD remain partially understood, with certain adverse reactions reported in clinical contexts. Consequently, obesity, weight loss, and insulin resistance emerge as current focal points, warranting further investigation. To ensure the diet's safe and standardized application, we recommend conducting more comprehensive clinical studies that delve deeper into its safety profile and underlying biological mechanisms.

## AUTHORS’ CONTRIBUTIONS

The authors confirm their contribution to the paper as follows: study conception and design: J.Z.; data collection: Y.J., Z.H.; data analysis and interpretation: T.C., Y.W.; writing the paper: X.Z. All authors reviewed the results and approved the final version of the manuscript.

## Figures and Tables

**Table 1:** Top 10 most globally cited articles.

**Paper**	**DOI**	**Total Citations**	**TC per Year**	**Normalized TC**
YANCY WS, 2004, ANN INTERN MED	10.7326/0003-4819-140-10-200405180-00006	612	30.60	4.33
DUSHAY J, 2010, GASTROENTEROLOGY	10.1053/j.gastro.2010.04.054	454	32.43	5.79
HOPKINS BD, 2018, NATURE	10.1038/s41586-018-0343-4	356	59.33	7.09
KENNEDY AR, 2007, AM J PHYSIOL-ENDOC M	10.1152/ajpendo.00717.2006	301	17.71	3.35
BADMAN MK, 2009, ENDOCRINOLOGY	10.1210/en.2009-0532	292	19.47	3.77
HESSION M, 2009, OBES REV	10.1111/j.1467-789X.2008.00518.x	269	17.93	3.47
NEWMAN JC, 2017, CELL METAB	10.1016/j.cmet.2017.08.004	256	36.57	4.61
FORSYTHE CE, 2008, LIPIDS	10.1007/s11745-007-3132-7	227	14.19	3.11
AUSTIN GL, 2011, AM J CLIN NUTR	10.3945/ajcn.110.000141	226	17.38	3.19
GOLDBERG EL, 2017, CELL REP	10.1016/j.celrep.2017.02.004	216	30.86	3.89

**Table 2:** Top 10 countries and institutions in terms of contribution.

**Rank**	**Country**	**Records**	**Institution**	**Records**
1	USA	169	UNIVERSITY OF NAPLES FEDERICO II	38
2	ITALY	89	SAPIENZA UNIVERSITY ROME	28
3	CHINA	38	UNIVERSITY OF ALABAMA BIRMINGHAM	24
4	SPAIN	25	DUKE UNIVERSITY	22
5	AUSTRALIA	21	HARVARD MEDICAL SCHOOL	20
6	JAPAN	19	OHIO STATE UNIVERSITY	20
7	GERMANY	15	UNIVERSITY OF TEXAS SYSTEM	20
8	CANADA	13	UNIVERSITY SYSTEM OF OHIO	18
9	UNITED KINGDOM	12	HARVARD UNIVERSITY	17
10	SWITZERLAND	11	UDICE-FRENCH RESEARCH UNIVERSITIES	17

**Table 3:** Top 10 authors under multiple influence indicators based on bibliometric package.

**Rank**	**Author**	**h-index**	**Author**	**g-index**	**Author**	**m-index**	**Author**	**Total Citation**
1	SAJOUX I	13	SAJOUX I	19	BARREA L	3	MARATOS-FLIER E	1674
2	CASANUEVA FF	11	CASANUEVA FF	14	SAJOUX I	1.3	WESTMAN EC	1271
3	CRUJEIRAS AB	11	CRUJEIRAS AB	13	CASANUEVA FF	1.1	SAJOUX I	761
4	WESTMAN EC	10	WESTMAN EC	12	CRUJEIRAS AB	1.1	CASANUEVA FF	693
5	MARATOS-FLIER E	10	MARATOS-FLIER E	10	BELLIDO D	0.9	CRUJEIRAS AB	634
6	BELLIDO D	9	VOLEK JS	10	CASTRO AI	0.857	VOLEK JS	603
7	VOLEK JS	8	BELLIDO D	9	COLAO A	0.75	BELLIDO D	574
8	COLAO A	6	COLAO A	9	MARATOS-FLIER E	0.588	CASTRO AI	278
9	BARREA L	6	BARREA L	8	WESTMAN EC	0.5	COLAO A	93
10	CASTRO AI	6	CASTRO AI	6	VOLEK JS	0.421	BARREA L	74

**Table 4:** Top 10 core sources by Bradford's law.

Journals	Rank	Number of Publications	JCR Partition	IF
Nutrients	1	47	1	5.9
Frontiers in Nutrition	2	15	1	5
American Journal of Physiology-Endocrinology and Metabolism	3	10	1	5.1
Nutrition	4	10	2	4.4
Nutrition & Metabolism	5	10	2	4.5
PLoS One	6	9	2	3.7
American Journal of Clinical Nutrition	7	8	1	7.1
Scientific Reports	8	8	2	4.6
Endocrine	9	7	3	3.7
Molecular Metabolism	10	7	1	8.1

**Table 5:** Top 25 keywords with the largest number of publications.

**Rank**	**Keywords**	**Counts**
1 2 3 4 5 6 7 8 9 10 11 12 13 14 15 16 17 18 19 20 21 22 23 24 25	obesity ketogenic diet weight-loss insulin-resistance low-fat diet metabolism low-carbohydrate body-composition management overweight inflammation glucose fat adults expression randomized-trial risk-factors ketone-bodies oxidative stress protein disease energy-expenditure high-fat appetite association	163 145 129 76 57 57 49 48 46 42 38 35 33 32 32 32 32 29 28 28 27 27 27 25 25

## Data Availability

The data supporting the findings of the article are available within the article.

## LIST OF ABBREVIATIONS

KBs: Ketone Bodies
βOHB: 3-β-hydroxybutyrate
MAD: Modified Atkins Diet
